# Radiotherapy treatment planning with contrast-enhanced computed tomography: feasibility of dual-energy virtual unenhanced imaging for improved dose calculations

**DOI:** 10.1186/1748-717X-9-168

**Published:** 2014-07-29

**Authors:** Sachiko Yamada, Takashi Ueguchi, Toshiyuki Ogata, Hirokazu Mizuno, Ryota Ogihara, Masahiko Koizumi, Takeshi Shimazu, Kenya Murase, Kazuhiko Ogawa

**Affiliations:** 1 Department of Radiology, Osaka University Hospital, 2-15 Yamadaoka, Suita, Osaka 565-0871, Japan; 2 Department of Medical Physics and Engineering, Osaka University Graduate School of Medicine, 1-7 Yamadaoka, Suita, Osaka 565-0871, Japan; 3 Center for Information and Neural Networks, National Institute of Information and Communications Technology, 1-4 Yamadaoka, Suita, Osaka 565-0871, Japan; 4 Graduate School of Frontier Biosciences, Osaka University, 1-4 Yamadaoka, Suita, Osaka 565-0871, Japan; 5 Kobe Minimally Invasive Cancer Center, 8-5-1 Minatojima-nakamachi, Chuo-ku, Hyogo 650–0046, Kobe, Japan; 6 Department of Traumatology and Acute Critical Medicine, Osaka University Graduate School of Medicine, 2-2 Yamadaoka, Suita, Osaka 565-0871, Japan; 7 Department of Radiation Oncology, Osaka University Graduate School of Medicine, 2-2 Yamadaoka, Suita, Osaka 565-0871, Japan

**Keywords:** Radiotherapy treatment planning, Dual-energy CT, Virtual unenhanced CT, Dose calculation, Iodine contrast agent, Tissue attenuation, CT number, Dual-source CT, Tin filter

## Abstract

**Background:**

In radiotherapy treatment planning, intravenous administration of an iodine-based contrast agent during computed tomography (CT) improves the accuracy of delineating target volumes. However, increased tissue attenuation resulting from the high atomic number of iodine may result in erroneous dose calculations because the contrast agent is absent during the actual procedure. The purpose of this proof-of-concept study was to present a novel framework to improve the accuracy of dose calculations using dual-energy virtual unenhanced CT in the presence of an iodine-based contrast agent.

**Methods:**

Simple phantom experiments were designed to assess the feasibility of the proposed concept. By utilizing a “second-generation” dual-source CT scanner equipped with a tin filter for improved spectral separation, four CT datasets were obtained using both a water phantom and an iodine phantom: “true unenhanced” images with attenuation values of 2 ± 11 Hounsfield Units (HU), “enhanced” images with attenuation values of 274 ± 23 HU, and two series of “virtual unenhanced” images synthesized from dual-energy scans of the iodine phantom, each with a different combination of tube voltages. Two series of virtual unenhanced images demonstrated attenuation values of 12 ± 29 HU (with 80 kVp/140 kVp) and 34 ± 10 HU (with 100 kVp/140 kVp) after removing the iodine component from the contrast-enhanced images. Dose distributions of the single photon beams calculated from the enhanced images and two series of virtual unenhanced images were compared to those from true unenhanced images as a reference.

**Results:**

The dose distributions obtained from both series of virtual unenhanced images were almost equivalent to that from the true unenhanced images, whereas the dose distribution obtained from the enhanced images indicated increased beam attenuation caused by the high attenuation characteristics of iodine. Compared to the reference dose distribution from the true unenhanced images, the dose distribution pass rates from both series of virtual unenhanced images were greater than 90%, while those from the enhanced images were less than approximately 50–60%.

**Conclusions:**

Dual-energy virtual unenhanced CT improves the accuracy of dose distributions in radiotherapy treatment planning by removing the iodine component from contrast-enhanced images.

## Background

The use of computed tomography (CT) is now an established procedure in modern radiotherapy treatment planning. Cross-sectional CT data allow for the accurate delineation of surface contours and internal structures including target and critical organs. In addition, the pixel values, i.e., the CT numbers in Hounsfield Units (HU), provide tissue electron density information, which allows a pixel-by-pixel correction for tissue inhomogeneities when calculating dose distributions. Although other imaging modalities are becoming important tools in treatment planning, CT is still the fundamental and primary modality.

An inherent disadvantage of CT is its relatively lower contrast between lesions and surrounding tissue or vessels. Thus, diagnostic CT often requires the intravenous administration of an iodine-based contrast agent to enhance lesion-to-tissue contrast. Because the accurate delineation of target volumes (and organs at risk) is crucial in radiotherapy treatment planning, the use of an iodine-based contrast agent is necessary in situations in which contrast is likely to improve the accuracy of the target or normal structure delineations. However, there is a concern that the introduction of contrast agent may result in erroneous dose calculations [1]. In an actual treatment situation, the patient does not receive contrast agent, and hence the tissue density during treatment will differ from that during a contrast-enhanced CT scan. Although several clinical studies on head and neck [2], lung [3], and prostate cancers [4] have shown that contrast media had little influence on dose calculations, this issue is still debated [5-7].

**Figure 1:**
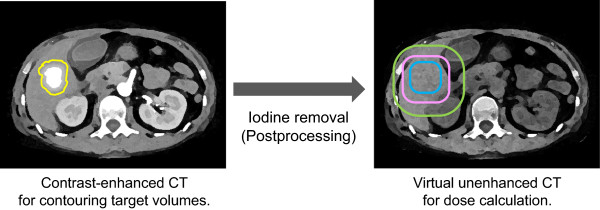
**Schematic of the proposed concept.** Dose calculations can be performed using synthesized virtual unenhanced CT images, while the delineation of the target and critical organs can be conducted using the original contrast-enhanced CT images.

A recent advance in the field of CT was the introduction of dual-energy CT. Dual-energy CT acquires two different datasets with different tube voltages (i.e., beam energies). Within the range of photon energies in CT, the CT numbers of soft tissues are almost independent of beam energy. In contrast, CT numbers of high atomic number materials such as iodine are noticeably energy-dependent. Thus, materials with a high atomic number can be further differentiated from soft tissues by applying different beam energies and analyzing the differences in attenuation [8]. This principle allows clinicians to reconstruct “virtual unenhanced” images from iodine contrast-enhanced scans [9-15]. The attenuation differences of iodine between two energies can be utilized to produce an “iodine map”, which can be subsequently removed from images to create virtual unenhanced images. Thus, virtual unenhanced images theoretically provide CT numbers equivalent to those of true unenhanced images. It should be noted that the key to reliable virtual unenhanced imaging lies on both the spectral separation between low- and high-energy beams, and low image noise. These two fundamental requirements can be accomplished by the “second generation” dual-source CT scanner. The scanner utilizes two distinct X-ray sources with corresponding detectors at an angle separation of 95°. The two sources can be simultaneously operated at different tube voltages. Importantly, this feature has the capability of applying an additional tin filter for a high-energy beam. The filter is highly advantageous because it narrows the spectrum, which results in higher dose efficiency, less beam hardening, and improved spectral separation between the low- and high-energy beams [16,17]. The improved spectral separation allows for tube operation at a combined 100 kVp and 140 kVp rather than the conventional dual-energy combination of 80 kVp and 140 kVp [17], and thus can reduce image noise. Furthermore, iodine removal is more accurate with the filter than without it when the subject adjoins a high attenuation material (e.g. bone, contrast-enhanced large vessels, etc.) [13].Using this promising technique under iodine contrast enhancement, we propose a novel strategy for improved radiation treatment planning. In this scheme, dose calculations can be performed using synthesized virtual unenhanced CT images, while the delineation of the target and critical organs can be conducted using the original contrast-enhanced CT images (Figure 1). The purpose of this preliminary study was to investigate the feasibility of our proposed scheme in simple phantom experiments using the second-generation dual-source CT scanner.

## Methods

This study comprised two phantom experiments. The first experiment assessed dual-energy virtual unenhanced attenuation values of iodine solutions under two different combinations of tube voltages. The second experiment investigated the feasibility of dose calculation using virtual unenhanced imaging. All experiments were conducted using a dual-source CT scanner (SOMATOM Definition Flash, Siemens AG, Erlangen, Germany). A dedicated application “Liver VNC” (Siemens) was utilized to reconstruct virtual unenhanced images.

### Assessment of virtual unenhanced attenuation values

We prepared a phantom comprising eight polypropylene syringes, each containing a 10-mL mixture of water and an iodine-based contrast agent (Iopamidol, 300 mgI/mL) at different dilutions (4.50%, 2.00%, 1.00%, 0.50%, 0.25%, 0.13%, 0.06%, and 0.00% by weight). The phantom was placed at the isocenter of the CT scanner, and scanned using both single-energy and dual-energy helical modes. For the single-energy scan, only one X-ray tube was operated at 120 kVp and 100 mAs. For the dual-energy scan, two combinations of tube settings, each using the tin filter for the high-energy beam, were tested: (i) 80 kVp with 100 mAs and 140 kVp with 39 mAs (referred to as the “80/Sn140-kVp protocol”); and (ii) 100 kVp with 100 mAs and 140 kVp with 77 mAs (referred to as the “100/Sn140-kVp protocol”). The slice thickness and reconstructed field-of-view for both scans were 5 mm and 150 mm, respectively. The images were reconstructed with standard body kernels (B35f for single-energy scans and D26f for dual-energy scans). Two series of virtual unenhanced images (i.e., 80/Sn140-kVp and 100/Sn140-kVp protocols) were synthesized from the corresponding dual-energy data. In addition, “mixed” images were synthesized using a linear weighting of the low- and high-kVp images. In diagnostic dual-energy applications, the mixed images are used as representative of dual-energy data, because they are similar to traditional single-energy (i.e., 120 kVp) images. Circular regions-of-interest (ROIs) were positioned within the syringes to obtain mean attenuation values in HU. The size of the ROIs was chosen to encompass an approximately 80% area of the syringes. The scans were repeated three times, and the mean attenuation values and standard deviation of the three measurements were calculated for comparisons.

### Feasibility of dose calculation using virtual unenhanced CT

**Figure 2:**
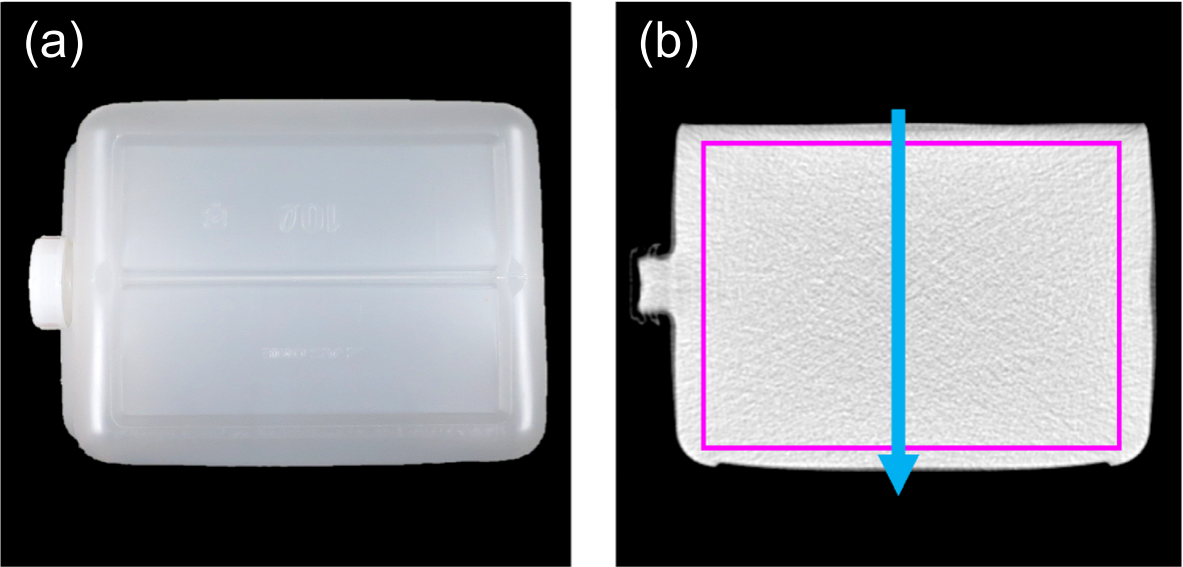
**The phantom used in the second experiment.** Photograph **(a)** and CT image **(b)** of the polypropylene container. The purple rectangle and blue arrow in **(b)** illustrate the region of interest for attenuation measurements and position of attenuation profile measurements, respectively.

Because this was a pilot study, a simple phantom experiment was designed to assess the feasibility of dose calculations on virtual unenhanced CT images. Two polypropylene containers (250 mm width, 175 mm height, and 350 mm depth) filled either with distilled water (referred to as the “water phantom”) or diluted (3%) Iopamidol contrast agent (referred to as the “iodine phantom”) were prepared and imaged to generate four CT datasets. First, the water phantom was scanned in single-energy mode, providing the “true unenhanced” images. Second, the iodine phantom was scanned in single-energy mode, providing the “enhanced” images. Third, the iodine phantom was scanned in dual-energy mode with the 80/Sn140-kVp protocol, providing the “virtual unenhanced (80/Sn140-kVp)” images. Finally, the iodine phantom was scanned in dual-energy mode with the 100/Sn140-kVp protocol, providing the “virtual unenhanced (100/Sn140-kVp)” images. Each phantom was positioned on the patient table and scanned at the isocenter of the CT scanner. The imaging parameters were the same as those described above, except that the slice thickness was 2.5 mm and the displayed field-of-view was 330 mm.Attenuation values of the phantoms depicted in the four datasets were measured using ROI analysis at the central slice. The mean and standard deviation of the attenuation values for each image were obtained from a rectangular ROI positioned to encompass greater than an 80% area of the phantom, but inside the outer edges. Furthermore, attenuation profiles passing from the top to the bottom of the phantom were obtained (adjacent ten profile curves were measured and then averaged to reduce fluctuations due to noise) to evaluate attenuation values outside the ROI (especially at the bottom of the phantom where iodine sedimentation is likely to occur), although we took care to uniformly dilute the contrast agent during preparation of the iodine phantom. Figure 2 shows a photograph of the phantom and its corresponding CT images, detailing both the ROI setting for attenuation measurements and the reference line for profile curve measurements.

These four CT datasets were transferred to a Pinnacle^3^ treatment planning system (version 8.0 m; Philips, Madison, WI, USA) for dose calculations. The dose distribution of a single photon beam from a Siemens Oncor Impression Plus system was calculated using a collapsed cone convolution algorithm with a dose grid size of 2 mm. Two beam energies (4 MV and 10 MV) and two field sizes (4 × 4 cm^2^ and 10 × 10 cm^2^) were tested for each dataset. The beam was planned to deliver 100 monitor units at a 10-cm depth at the center of the phantom. Although the phantom was carefully prepared, it was impossible to avoid introducing small air bubbles at the top of the phantom. Therefore, the posterior field was chosen to minimize the effects of these small bubbles on the dose calculations. The dose distributions obtained from the enhanced and virtual unenhanced (both the 80/Sn140-kVp and 100/Sn140-kVp protocols) images were quantitatively compared to those from the true unenhanced images using MapCHECK (Sun Nuclear, Melbourne, FL, USA) analysis software. For the analysis, the calculated planar dose distribution in the sagittal on-axis plane for each dataset was imported into the software; the distribution obtained from the true unenhanced images was used as a reference, and the remaining three were the subjects being tested. The assessment was performed based on the percent-dose-difference and distance-to-agreement at a 10% dose threshold. A set of acceptance criteria (2% and 2 mm) satisfying the guideline [18] was used, and the percentage of points passing the acceptance criteria was evaluated.

## Results

**Figure 3:**
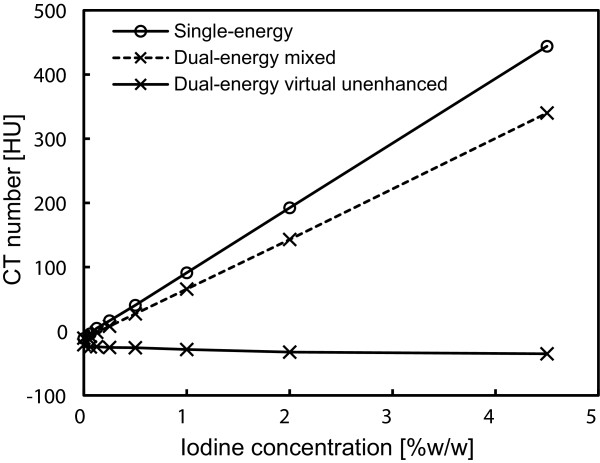
**Attenuation measurements of iodine solutions at different iodine concentrations.** Single-energy images were acquired at a tube voltage of 120 kVp. Mixed images and virtual unenhanced images were synthesized from dual-energy (100 kVp and 140 kVp with a tin filter) data.

Figure 3 shows the results of attenuation measurements for the iodine syringes on the single-energy, and the dual-energy (100/Sn140-kVp protocol) mixed and virtual unenhanced images. The exact attenuation values including both the 80/Sn140-kVp and 100/Sn140-kVp protocols are listed in Table 1. A range of iodine concentrations tested in this experiment provided attenuation values up to approximately 440 HU under the single-energy scan. The virtual unenhanced attenuation values using this range of iodine concentrations were reasonably stable and were close to both the single-energy and dual-energy mixed attenuation values at a 0% iodine concentration.

**Table 1:** Attenuation measurements of iodine solutions at different iodine concentrations

**Iodine concentration [%w/w]**	**Attenuation value [HU]**				
	**Single-energy (120 kVp)**	**Dual-energy (80 kVp/Sn140 kVp)**		**Dual-energy (100 kVp/Sn140 kVp)**	
		**Mixed**	**Virtual unenhanced**	**Mixed**	**Virtual unenhanced**
0.00	-11.3 ± 1.3	-10.4 ± 0.1	-23.0 ± 1.0	-10.7 ± 0.6	-21.0 ± 0.6
0.06	-3.9 ± 0.5	-6.9 ± 0.1	-26.8 ± 0.9	-8.0 ± 0.4	-24.5 ± 1.5
0.13	4.7 ± 0.5	0.1 ± 0.2	-25.1 ± 0.8	-1.6 ± 0.4	-24.1 ± 0.7
0.25	16.4 ± 0.4	11.1 ± 1.1	-26.6 ± 1.8	7.9 ± 0.8	-25.3 ± 1.1
0.50	40.5 ± 0.5	33.5 ± 0.2	-25.8 ± 1.1	27.2 ± 0.4	-25.7 ± 1.1
1.00	91.1 ± 0.4	78.5 ± 0.4	-27.1 ± 1.1	65.8 ± 0.6	-28.4 ± 0.4
2.00	192.3 ± 0.3	168.7 ± 0.3	-29.1 ± 0.3	143.0 ± 0.3	-32.5 ± 1.3
4.50	444.1 ± 0.7	397.9 ± 0.4	-28.7 ± 0.3	340.1 ± 0.3	-35.1 ± 1.9

Note: Attenuation values are mean ± standard deviation from three measurements.

**Figure 4:**
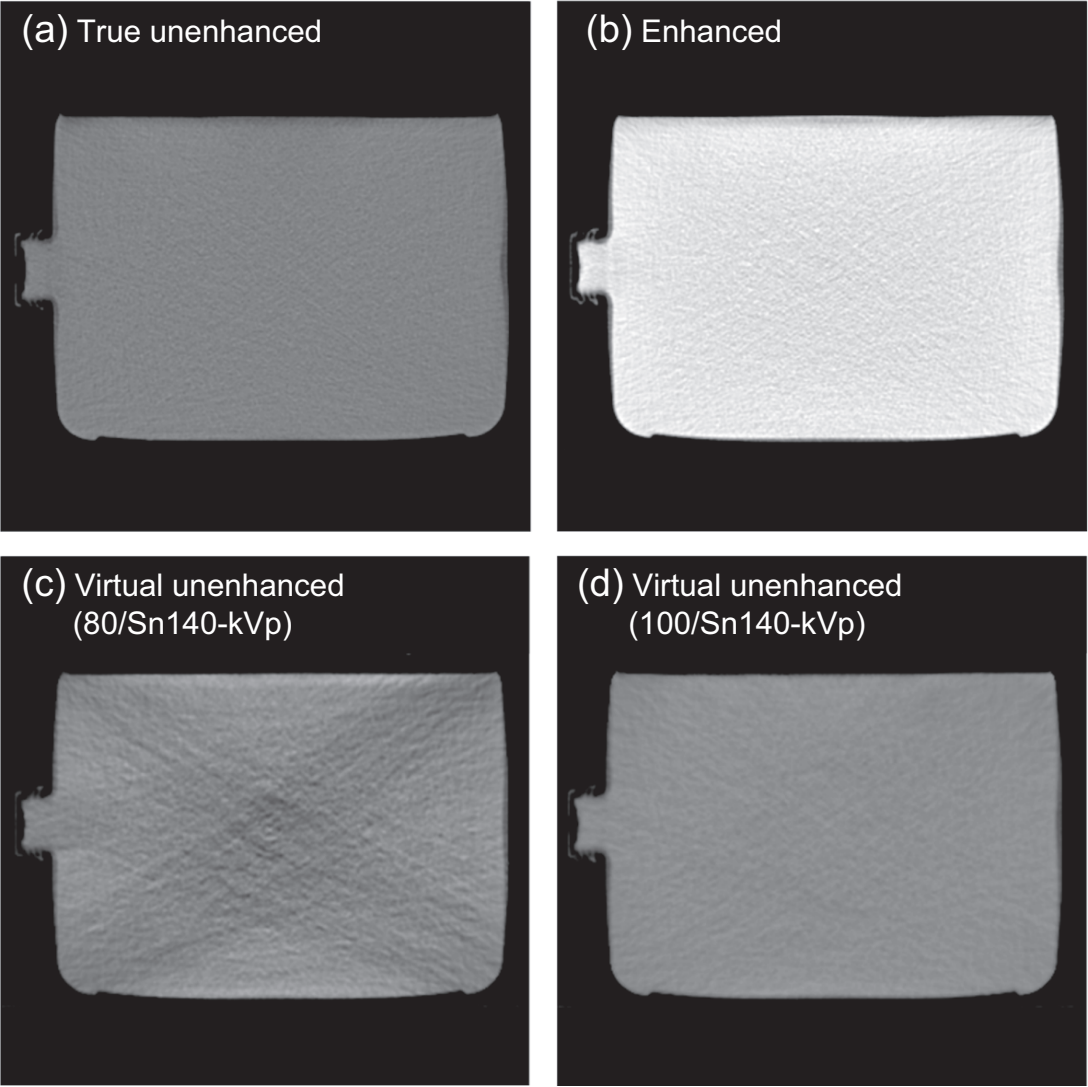
**Representative CT images of the phantom for dose calculations.** True unenhanced images **(a)** were obtained using the water phantom. Remaining enhanced images **(b)**, virtual unenhanced (80/Sn140-kVp) images **(c)**, and virtual unenhanced (100/Sn140-kVp) images **(d)** were obtained using the iodine phantom. The enhanced image **(b)** shows almost uniform attenuation, indicating no obvious iodine sedimentation. The virtual unenhanced image obtained using the 80/Sn140-kVp protocol shows dark-band artifact and increased image noise, while the image using the 100/Sn140-kVp protocol provided adequate image quality.

**Figure 5:**
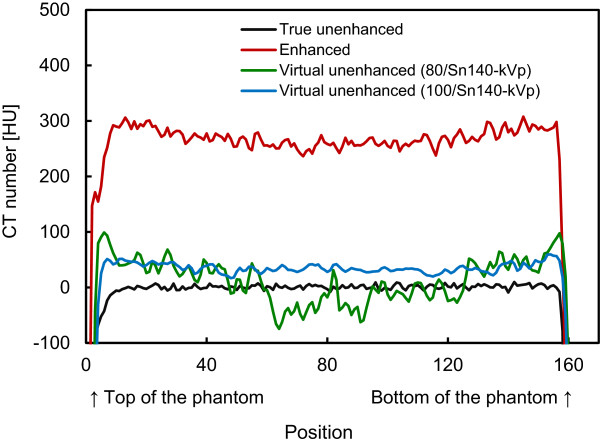
**Attenuation profiles.** Attenuation values in HU are plotted against the pixel locations (shown in Figure 2b). The profile from the enhanced image (red line) shows almost uniform distribution of attenuation values with a slight cupping due to beam hardening, indicating no obvious iodine sedimentation. The profile from the virtual unenhanced images using the 100/Sn140-kVp protocol is quite stable, while the profile using the 80/Sn140-kVp protocol was highly fluctuated by dark-band artifacts.

Figure 4 shows representative (a) true unenhanced, (b) enhanced, (c) virtual unenhanced (80/Sn140-kVp), and (d) virtual unenhanced (100/Sn140-kVp) images of the phantom. All the CT images were displayed at fixed window settings (window level, 50 HU; window width, 600 HU), allowing us to compare the image contrast between the four images, visualize the effect of tube voltage settings on virtual unenhanced images (c and d), and visually confirm the presence or absence of iodine sedimentation (b). The virtual unenhanced image with the 80/Sn140-kVp protocol showed higher image noise and dark-band artifact, while the image obtained with the 100/Sn140-kVp protocol showed more uniform attenuation and less image noise. No obvious iodine sedimentation was found in the enhanced image (b). Figure 5 shows the attenuation profiles of the phantom. The profile from the enhanced image (red line) indicates that the iodine was almost uniformly distributed inside the phantom because the profile is almost flat, although the profile was slightly cupped due to beam hardening. The attenuation values of the phantoms depicted in the true unenhanced, enhanced, and virtual unenhanced images are listed in Table 2. The mean attenuation values of the iodine phantom on the virtual unenhanced images were lower (11.6 ± 29.2 HU with the 80/Sn140-kVp protocol and 34.1 ± 9.9 HU with the 100/Sn140-kVp protocol) than those on the enhanced image (274.3 ± 22.5 HU).

**Table 2:** Attenuation measurements of water and iodine phantoms

**Measurements**	**Attenuation values [HU]**
Water phantom/true unenhanced image	1.6±11.0
Iodine phantom/enhanced image	274.3±22.5
Iodine phantom/virtual unenhanced (80/Sn140-kVp) image	11.6±29.2
Iodine phantom/virtual unenhanced (100/Sn140-kVp) image	34.1±9.9

Note: Attenuation values are mean ± standard deviation in the region of interest.

**Figure 6:**
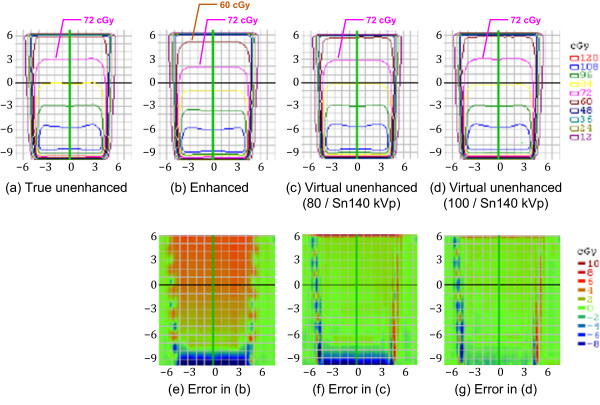
**Planar dose distributions and error maps (energy, 10 MV; field size, 10 × 10 cm**^**2**^**).** Dose calculations were performed using **(a)** true unenhanced, **(b)** enhanced, **(c)** virtual unenhanced (80/Sn140-kVp), and **(d)** virtual unenhanced (100/Sn140-kVp) images. The three error (difference) maps correspond to the **(e)** enhanced, **(f)** virtual unenhanced (80/Sn140-kVp), and **(g)** virtual unenhanced (100/Sn140-kVp) images, with the true unenhanced images as the reference. Dose distributions obtained from the virtual unenhanced images are quite similar to dose distributions from the true unenhanced images. In particular, the virtual unenhanced (100/Sn140-kVp) images achieved almost entirely 0-cGy of errors, except at the beam edges.

**Figure 7:**
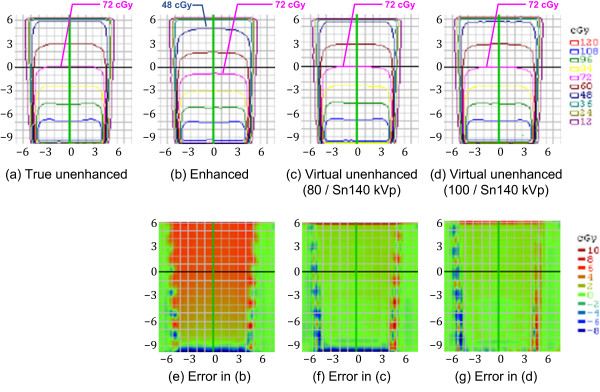
**Planar dose distributions and error maps (energy, 4 MV; field size, 10 × 10 cm**^**2**^**).** Dose calculations were performed using **(a)** true unenhanced, **(b)** enhanced, **(c)** virtual unenhanced (80/Sn140-kVp), and **(d)** virtual unenhanced (100/Sn140-kVp) images. The three error (difference) maps correspond to **(e)** enhanced, **(f)** virtual unenhanced (80/Sn140-kVp), and **(g)** virtual unenhanced (100/Sn140-kVp) images, with the true unenhanced images as the reference. In addition to findings similar to those in Figure 6, more prominent dose errors are elicited using the enhanced images **(e)**.

**Figure 8:**
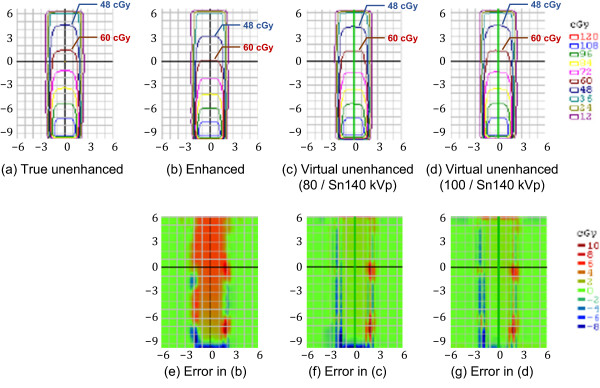
**Planar dose distributions and error maps (energy, 4 MV; field size, 4 × 4 cm**^**2**^**).** Dose calculations were performed using **(a)** true unenhanced, **(b)** enhanced, **(c)** virtual unenhanced (80/Sn140-kVp), and **(d)** virtual unenhanced (100/Sn140-kVp) images. The three error (difference) maps correspond to **(e)** enhanced, **(f)** virtual unenhanced (80/Sn140-kVp), and **(g)** virtual unenhanced (100/Sn140-kVp) images, with the true unenhanced images as the reference. Findings similar to those in Figures 6 and 7 were observed.

Figures 6, 7 and 8 compare the four representative planar dose distributions (a–d), planned at beam energies of either 10 MV (Figure 6) or 4 MV (Figures 7 and 8), and field sizes of either 10 × 10 cm^2^ (Figures 6 and 7) or 4 × 4 cm^2^ (Figure 8), obtained from the true unenhanced, enhanced, and virtual unenhanced (both the 80/Sn140-kVp and 100/Sn140-kVp protocols) CT images. The dose difference (i.e. error) maps derived between the tested (enhanced or virtual unenhanced) and reference (true unenhanced) dose distributions are also shown (e–g). In each condition (Figures 6, 7 and 8), the dose distributions obtained from both the virtual unenhanced images were quite similar to that generated from the true unenhanced images, whereas the dose distribution obtained from the enhanced image indicated increased beam attenuation. Using the enhanced images, errors in dose calculations increased as the beam passed from the bottom to the top of the iodine phantom. In contrast, the errors were almost entirely 0 cGy when the virtual unenhanced images, especially the 100/Sn140-kVp protocol, were used. Table 3 compares the pass rates of the dose calculations based on the virtual unenhanced and enhanced images. When the virtual unenhanced images were utilized for dose calculations, pass rates greater than 90% were achieved, while the pass rates were less than approximately 50–60% when enhanced images were used.

**Table 3:** Pass rates of dose calculations based on the enhanced and virtual unenhanced CT images

**Energy [MV]**	**Field size [cm**^**2**^**]**	**Pass rate [%]**		
		**Enhanced**	**Virtual unenhanced**	
			**80 kVp/Sn140 kVp**	**100 kVp/Sn140 kVp**
10	10 × 10	45.6	98.4	98.4
10	4 × 4	59.3	98.4	99.2
4	10 × 10	37.1	93.7	97.9
4	4 × 4	44.9	98.7	98.4

## Discussion

The use of a contrast agent during CT for treatment planning offers the potential benefit of a much higher accuracy in delineating target and critical volumes. However, there is a significant concern that the high atomic number of iodine may introduce errors into dose calculations; the calculated dose may be underestimated, resulting in the delivery of a higher dose than prescribed. This scenario would occur in organs containing an increased iodine concentration. In fact, Shibamoto et al. found that the administration of a contrast agent influenced treatment planning at the upper abdomen, especially when the beams passed through organs with high iodine concentrations such as the liver, spleen, and kidneys [5]. Our present results support their findings. The iodine phantom used in this study presented almost uniformly distributed attenuation values of approximately 270 HU (Figure 4b; Figure 5; Table 2), which approximates that of organs in the upper abdomen on contrast-enhanced CT. As shown in Figures 6, 7 and 8(b), the dose distributions obtained from the enhanced images show increased beam attenuation compared to those from the true unenhanced images (a). The corresponding error maps (Figures 6, 7 and 8e) show that the calculated dose obtained from the enhanced images tends to differ from that based on the true unenhanced images as the beam passes through the phantom from the bottom to the top. In Figure 7, for instance, the dose distribution (a) obtained using the true unenhanced images shows a delivery of 72 cGy at the 10-cm depth of the central axis; in contrast, the dose error was at least 4 cGy when the enhanced images were used (e). In this case, the use of the enhanced images leads to erroneous dose delivery greater than 5% of the prescribed dose. This error is not negligible and would be problematic for treatment planning, especially in the upper abdomen as Shibamoto et al. reported [5].

Our novel approach to solve this issue is to remove the iodine component from contrast-enhanced CT using dual-energy virtual unenhanced imaging. Dual-energy virtual unenhanced imaging has recently become a promising technique in diagnostic CT to reduce the radiation dose by negating the need for an unenhanced scan [9-11]. Although the clinical potential of dual-energy CT in the field of radiation oncology has not been well investigated to date, several studies have shown that dual-energy CT allows for an accurate estimation of electron density for treatment planning [19-21]. Our study reveals an additional important aspect of its application that could improve the accuracy of radiotherapy treatment planning. Figure 3 shows that virtual unenhanced CT can remove the iodine component from a contrast-enhanced CT image within a wide range of iodine concentrations at CT number levels greater than the water density, in accordance with previous reports [9-14], including a similar phantom study of iodine solutions [10]. Thus, our study aimed to improve the accuracy of dose calculations from contrast-enhanced CT by removing their iodine components, and the results successfully demonstrated the feasibility of our proposed method. As shown in Figures 6, 7 and 8, the dose distributions obtained from virtual unenhanced images (c and d) were almost equivalent to those from true unenhanced images (a). The differences in dose calculations between true unenhanced and virtual unenhanced images were extremely close to 0 cGy (f and g), except at the beam edges and dose build-up regions (near the bottom of the phantom), which both have high dose gradients. In addition, the use of the 100/Sn140-kVp protocol further improved the accuracy of dose distributions at the dose build-up region. The pass rates of dose calculations (Table 3) from the virtual unenhanced images were greater than 90%, whereas those from the enhanced images were less than approximately 50–60% and thus no longer acceptable.

The virtual unenhanced imaging results in potentially unstable CT numbers when the differences between attenuation values at low- and high-kVp are small. Therefore, the “first-generation” dual-source CT scanner minimized spectral overlap by performing the low-energy scan at 80 kVp [8]. However, the use of an 80-kVp beam brought about an inappropriately low photon rate at the detectors, especially in the abdominal scan, due to a low penetration. Thus, the resultant virtual unenhanced images suffered from increased image noise and artifacts. Although the tin filter could improve spectral separation, the use of an 80-kVp beam would still be problematic. The virtual unenhanced attenuation values of iodine syringes with the 80/Sn140-kVp protocol were indeed more stable than those obtained with the 100/Sn140-kVp protocol at high iodine concentrations (Table 1); this experiment was performed with a small phantom. In fact, the virtual unenhanced (80/Sn140-kVp) images of the iodine phantom, which was used in the second experiment, suffer from increased noise and dark-band artifacts (Figure 4,c). The attenuation profile with the 80/Sn140-kVp protocol (Figure 5, green line) fluctuated greatly at the center of the phantom, due to the dark-band artifacts. In contrast, the virtual unenhanced attenuation values using the 100/Sn140-kVp protocol were stable qualitatively (Figure 4d) and quantitatively (Figure 5, blue line). The mean attenuation values of the virtual unenhanced (100/Sn140-kVp) and true unenhanced images were 34.1 ± 9.9 HU and 1.6 ± 11.0 HU, respectively. This small difference had no effect on dose calculations as shown in Figures 6, 7 and 8.

The current dual-source virtual unenhanced CT has several known potential limitations, which were investigated by Barrett, et al. [9]. For radiotherapy treatment planning, its relatively smaller field-of-view and susceptibility to motion warrants consideration. The effective field-of-view that can remove iodine components from contrast-enhanced images is limited to 322 mm or less in diameter. This is problematic in larger patients or in more superficial radiotherapy targets because the contrast-enhanced tissue that is located outside the region will remain “enhanced” after the iodine-removal process. This limitation arises from the current scanner configuration, and therefore, it can be alleviated in the future. Also notable is the relatively higher susceptibility to motion in virtual unenhanced imaging, which arises from the 95° offset of the source-detector systems. The angle offset causes one detector to see a different motion compared to the other detector, and this difference is amplified by the subtraction process. A shorter gantry rotation time can reduce motion artifacts, but the potential for the artifact remains, particularly in the upper-abdominal organs, lung bases, and cardiovascular systems. Another potential limitation of virtual unenhanced imaging is the underestimation of attenuation values of high atomic number materials such as bone. In previous reports [9-11,14], clinical virtual unenhanced images visually demonstrated highly attenuating bone, resembling the normal CT appearance; the iodine removal process would affect the virtual unenhanced attenuation value, depending on the parameters of iodine removal (e.g., “standard tissue” and “fat” CT numbers, “beam-hardening correction” option for iodine, and “relative contrast enhancement ratio” value between low- and high-energy images in “Liver VNC” application). We expect that this underestimation of bone attenuation has little influence on the dose calculation because bone (especially cortical bone) encompasses a relatively small volume within the trunk; however, further investigations evaluating the accuracy and the impact of bone virtual unenhanced attenuation values on dose calculations, optimizing the processing parameters, and (if necessary) developing a correction scheme are needed.

This proof-of-concept study is very preliminary, and therefore has several limitations. First, we only conducted simple phantom experiments and did not perform any clinical investigations. Second, the optimal acquisition and reconstruction parameters suitable for applying virtual unenhanced images to radiotherapy treatment planning were not investigated. These limitations are practical in nature; the dual-energy CT scanner used in this study is currently not available for radiotherapy treatment planning. Third, as mentioned above, the reliability of virtual unenhanced attenuation values of high atomic number materials such as bone has not been investigated. Further investigation of these issues is needed to confirm the clinical potential of virtual unenhanced CT in radiotherapy treatment planning.

## Conclusions

We have proposed a novel strategy to improve the accuracy of radiotherapy treatment planning using dual-energy virtual unenhanced CT. The introduction of contrast agents can improve the delineation of the target and risk organs. The iodine component can be subsequently removed from contrast-enhanced CT images to provide virtual unenhanced images to perform more accurate dose calculations. Our results have successfully provided the first proof-of-concept.

## Competing interests

The authors declare that they have no competing interests.

## Authors’ contributions

SY conceived of the study, participated in its design and coordination, and drafted the manuscript. SY, TU, TO, HM, RO, MK, TS, and KO contributed to data acquisition. SY, TU, TO, HM, MK, KM, and KO performed data analysis and interpretation. TU, TO, HM, RO, MK, TS, KM, and KO helped to finalize the manuscript. All authors read and approved the final manuscript.
